# Clonal in vitro propagation of peat mosses (*Sphagnum* L.) as novel green resources for basic and applied research

**DOI:** 10.1007/s11240-014-0658-2

**Published:** 2014-11-14

**Authors:** Anna K. Beike, Valeria Spagnuolo, Volker Lüth, Feray Steinhart, Julia Ramos-Gómez, Matthias Krebs, Paola Adamo, Ana Isabel Rey-Asensio, J. Angel Fernández, Simonetta Giordano, Eva L. Decker, Ralf Reski

**Affiliations:** 1 Plant Biotechnology, Faculty of Biology, University of Freiburg, Schänzlestraße 1, 79104 Freiburg, Germany; 2 Dipartimento di Biologia, Università di Napoli Federico II, Campus Monte S. Angelo, Via Cinthia 4, 80126 Naples, Italy; 3 Department of Cellular Biology and Ecology, Faculty of Biology, University of Santiago de Compostela, 15782 Santiago de Compostela, Spain; 4 BIOVIA Consultor Ambiental, Edificio Emprendia, Campus Vida, 15782 Santiago de Compostela, Spain; 5 Institute of Botany and Landscape Ecology, Ernst-Moritz-Arndt University of Greifswald, 17487 Greifswald, Germany; 6 Dipartimento di Agraria, Università di Napoli Federico II, Via Università 100, 80055 Naples, Italy; 7 AMRA S.c.a r.l., Via Nuova Agnano 11, 80125 Naples, Italy; 8 BIOSS - Centre for Biological Signalling Studies, Freiburg, Germany; 9 FRIAS - Freiburg Institute for Advanced Studies, Freiburg, Germany

**Keywords:** Biomonitoring, In vitro cultivation, Moss bag technique, Mosses, *Sphagnum*, *Sphagnum* propagule

## Abstract

As builders and major components of peatlands, *Sphagnopsida* (peat mosses) are very important organisms for ecosystems and world’s climate. Nowadays many *Sphagnum* species as well as their habitats are largely protected, while their scientific and economic relevance remains considerable. Advanced methods of in vitro cultivation provide the potential to work in a sustainable way with peat mosses and address aspects of basic research as well as biotechnological and economical topics like biomonitoring or the production of renewable substrates for horticulture (*Sphagnum* farming). Here, we describe the establishment of axenic in vitro cultures of the five peat moss species *Sphagnum fimbriatum* Wils. and Hook., *Sphagnum magellanicum* Brid., *Sphagnum palustre* L., *Sphagnum rubellum* Wils. and *Sphagnum subnitens* Russ. and Warnst. with specific focus on large-scale cultivation of *S. palustre* in bioreactors. Axenic, clonal cultures were established to produce high quantities of biomass under standardized laboratory conditions. For advanced production of *S.* *palustre* we tested different cultivation techniques, growth media and inocula, and analyzed the effects of tissue disruption. While cultivation on solid medium is suitable for long term storage, submerse cultivation in liquid medium yielded highest amounts of biomass. By addition of sucrose and ammonium nitrate we were able to increase the biomass by around 10- to 30-fold within 4 weeks. The morphology of in vitro-cultivated gametophores showed similar phenotypic characteristics compared to material from the field. Thus the tested culture techniques are suitable to produce *S. palustre* material for basic and applied research.

**Electronic supplementary material:**

The online version of this article (doi:10.1007/s11240-014-0658-2) contains supplementary material, which is available to authorized users.

## Introduction

Peat mosses (*Sphagnopsida*) are a class of mosses (Bryophyta *sensu stricto*) with unique morphological, developmental and physiological characteristics (cf. Rydin and Jeglum 2013). As major components of peatlands they play an extensive role in earth’s ecosystems and climate (cf. Gorham 1991). Peatlands are an important sink of carbon sequestrated in peat (Joosten and Clarke 2002). Significant part of ecosystem carbon uptake is carried out by peat mosses, which cover over 1.5 million km^2^ (e.g. Street et al. 2013, Rydin and Jeglum 2013). Carbon fixed in living and dead plant material of *Sphagnum* is probably greater than fixed by terrestrial vegetation within 1 year and greater than of any other plant genus (Clymo and Hayward 1982). Regarding peatland ecology, *Sphagnopsida* are real ecosystem engineers as they acidify their surrounding habitat, thus creating conditions unsuitable for many competitive plants (Jones et al. 1994; van Breemen 1995). Besides their ecological relevance, peat mosses possess substantial economic relevance as represented by their utilization as substrates for horticulture (Johnson and Maly 1998; Whinam et al. 2003). While still collected from natural peatlands, promising research focuses on the cultivation of peat mosses on degraded peatlands to produce *Sphagnum* biomass, called *Sphagnum* farming (Gaudig et al. 2014) with an increasing application range (Oberpaur et al. 2010; Reinikainen et al. 2012; Blievernicht et al. 2013). Peat mosses are also used as packing, insulating or filtration material (e.g. Thieret 1956; Shaw et al. 2003). Furthermore, *Sphagnum* species are highly suitable as biomonitors for the assessment of air quality. Mosses can accumulate a variety of atmospheric pollutants including nitrogen compounds, organic compounds such as polycyclic aromatic hydrocarbons, radionuclides, metals and metalloids. The concentration in their tissues provides insights into air pollution at an area of interest (e.g. Thomas et al. 1984; Frahm 1998; Adamo et al. 2003; Solga et al. 2006; Giordano et al. 2013). A common technique for active biomonitoring with these plants is called “moss bag technique” (for review Ares et al. 2012), in which moss material is transferred to inert mesh bags and exposed to different areas of interest. The principle was developed already in the early seventies (Goodman and Roberts 1971). Typical species used for this purpose belong to the genus *Sphagnum* (Wegener et al. 1992; Vingiani et al. 2004), followed by pleurocarpous mosses like *Hylocomium splendens* (Hedw.) Schimp., *Hypnum cupressiforme* Hedw., *Pleurozium schreberi* Brid. (Mitt.) or *Pseudoscleropodium purum* (Hedw.) M. Fleisch. (Solga et al. 2006; Saxena et al. 2008; Ares et al. 2011). However, in comparison to other mosses, *Sphagnum* shows highest metal adsorption capacity while being most stable in terms of biomass degradation (González and Pokrovsky 2014).

Considering these aspects, it is evident that *Sphagnopsida* are important plants for basic as well as applied research. Recently, the United States Department of Energy Joint Genome Institute accepted a proposal to sequence the genome of a representative *Sphagnum* species for questions of carbon cycling and climate change (J. Shaw and D. Weston, PIs). As we have previously seen for the model organism *Physcomitrella patens* (Hedw.) Bruch and Schimp., the availability of the genome sequence extends scientific impact and research possibilities enormously (Rensing et al. 2008; Zimmer et al. 2013). For *P. patens* there are already well-established methods available, ranging from standardized in vitro cultivation to large-scale transcriptomic, proteomic and metabolomic analyses that provide novel insights into moss and thereby land plant evolution (e.g. Beike et al. 2014a, b; Mueller et al. 2014). However, as mosses comprise around 13,000 species (Goffinet et al. 2008) with enormous diversity, this forms just a basis within *Bryopsida*, not necessarily to speak of *Sphagnopsida*.

*Sphagnopsida* are in many aspects quite different from other mosses, a fact that makes them also relevant for evolutionary-developmental (evo-devo) studies. *Sphagnum* gametophores develop from thalloid protonema and have a strictly determined organography with unlimited apical growth (Clymo and Duckett 1986; Ligrone and Duckett 1998). Stem elongation involves apical as well as subapical meristematic activity, which appears to be unique among mosses (Ligrone and Duckett 1998). As recently published for *P. patens*, there are at least eight types of stem cells that determine its life cycle (Kofuji and Hasebe 2014). For *Sphagnopsida* this might be different, but it is to our knowledge not yet analyzed in more detail. Considering the phylogenetic position of peat mosses, the study of their stem cells would be only one topic that promises novel insights into stem cell evolution.

Such molecular evo-devo studies are facilitated by standardized methods of axenic in vitro cultivation. Starting with Becquerel (1906) who described the cultivation of *Atrichum undulatum* (Hedw.) P.Beauv. and *Hypnum velutinum* Hedw., axenic in vitro cultivation of Bryophyta (*sensu lato*), including liverworts, mosses and hornworts, has been constantly optimized (Duckett et al. 2004; Hohe and Reski 2005a; Beike et al. 2010). The establishment of photobioreactors further enhanced the opportunities for biotechnological applications, especially for *P. patens* which is nowadays e.g. used as production platform for the production of biopharmaceuticals (Hohe and Reski 2005b; Decker and Reski 2008).

The availability of *Sphagnum* species is limited as most species are rare in Western and Central Europe and protected e.g. via the European Council Habitat Directive (92/43/EEC) or the Fauna-Flora-Habitat Directive (92/43/EWG). *Sphagnopsida* in a scalable, standardized in vitro culture like in photobioreactors can enhance their scientific potential. Culture techniques with high multiplication rate of *Sphagnum* material is also of economic interest as *Sphagnum* farming depends on the availability of sufficient peat mosses as ‘seeding’ material for installation of *Sphagnum* cultures on degraded peatlands.

In this work, we established five *Sphagnum* species, namely *Sphagnum fimbriatum* Wils. and Hook., *Sphagnum magellanicum* Brid., *Sphagnum palustre* L., *Sphagnum rubellum* Wils. and *Sphagnum subnitens* Russ. and Warnst. in axenic in vitro culture and optimized the cultivation conditions for *S. palustre* towards biomass production. Starting from surface-sterilized spores, clonal propagation for all five species was achieved. For *S. palustre*, we propose different cultivation techniques, ranging from cultivation on solid medium in Petri dishes to advanced cultivation in bioreactors, and describe growth conditions for standardized large-scale production. As part of the European FP7 project “MOSSCLONE”, focusing on a standardization of the “moss bag technique” for biomonitoring, the *S. palustre* biomass will be analyzed with regard to their suitability for active biomonitoring. The combination of both, standardized cultivation and application, can improve the “moss bag technique” towards a highly reproducible and potentially cost-effective alternative to the use of automatic measuring devices.

## Materials and methods

### Collection of moss species and sterilization of spores

Sporangia from five *Sphagnum* species were collected in the field. For spore sterilization, mature capsules were transferred to 600 µL 0.1 % sodium hypochlorite (Merck, Darmstadt, Germany) solution and opened with sterile forceps by squeezing. Sodium hypochlorite solution was prepared freshly and 1 drop of Tween^®^20 (Merck, Darmstadt, Germany) was added per 10 mL of the solution. After incubation series of 45 s, 1, 1.5, 2, 2.5, 3, 3.5 and 4 min each 75 µL of the mixture were transferred to 4 mL autoclaved water. From this dilution 1 mL was transferred to a sterile Petri dish containing solid Knop medium (1.84 mM KH_2_PO_4_, 3.35 mM KCl, 1.01 mM MgSO_4_ * 7 H_2_O, 4.24 mM Ca(NO_3_)_2_ * 4 H_2_O, 45 µM FeSO_4_ * 7 H_2_O) according to Reski and Abel (1985). The Petri dishes were enclosed with Parafilm^®^ (Carl Roth GmbH, Karlsruhe, Germany) and kept under growth conditions of 70 μmol m^−2^ s^−1^ light intensity (Philips TLD 36 W/33-640) and a photoperiod of 16 h light to 8 h dark at 23 °C. After spore germination, single thalloid protonemata were transferred to new Petri dishes containing solid Knop medium. The transfer was done under sterile conditions using needles and a stereo microscope (Stemi 2000-C, Zeiss, Jena, Germany). For sterility control a swap with a needle was done, once on LB medium (10 g/L Bacto-Trypton (Becton, Dickinson and Company, Le Pont de Claix, France), 10 g/L NaCl, 5 g/L Bacto Yeast Extract (Becton, Dickinson and Company, Le Pont de Claix, France)), and once on Knop medium supplemented with 1 % glucose. The sterile controls were kept for at least 4 weeks at room temperature.

### In vitro cultivation techniques for *Sphagnum palustre* propagation

For cultivation of single clones of *S. palustre* on solid medium, gametophores that developed from thalloid protonema were transferred to solid Knop medium supplemented with microelements (50 µM H_3_BO_3_, 50 µM MnSO_4_ · 1 H_2_O, 15 µM ZnSO_4_ · 7 H_2_O, 2.5 µM KJ, 500 nM Na_2_MoO_4_ · 2 H_2_O, 50 nM CuSO_4_ · 5 H_2_O, 50 nM Co(NO_3_)_2_ · 6 H_2_O) according to Schween et al. (2003). The Petri dishes were enclosed either with Parafilm^®^ only (Carl Roth GmbH, Karlsruhe, Germany) or with micropore™ (VWR International GmbH, Darmstadt, Germany) covered with Parafilm^®^.

For cultivation of *S. palustre* clones in liquid medium, gametophores were transferred to Erlenmeyer flasks filled with 50 or 200 mL liquid medium, respectively, or to aerated round-bottom flasks containing 5 L liquid medium. For standard cultivation in flasks, liquid Knop medium supplemented with microelements (ME), 0.3 % sucrose and 1.25 mM ammonium nitrate (NH_4_NO_3_) was used. For large scale production in bioreactors, sucrose concentration was increased to 2 %. The positive effect of sucrose and NH_4_NO_3_ for cultivation of peat moss, namely *Sphagnum fallax* and *Sphagnum nemoreum*, had previously been described by Simola (1969, 1975) and Rudolph et al. (1988). The pH of the medium was adjusted to 4.8 with KOH and HCl before autoclaving. Ammonium nitrate solution was sterile filtered and added after autoclaving. The pH was measured using a pH electrode (pH 197-S, WTW GmbH, Weilheim, Germany). After autoclaving, a previously adjusted pH of 4.8 decreased to 4.1 (±0.1, n = 3), while a pH of 2.8 stayed at 2.8 (±0.03, n = 3), a pH of 3.8 decreased to 3.6 (±0.03, n = 3) and a pH of 5.8 decreased to 5.2 (±0.2, n = 3). In the following, the pH before autoclaving is described.

After transfer of *S. palustre* gametophores to liquid medium, the flasks were enclosed with silicone sponge closures (Hirschmann, Eberstadt, Germany). The suspension cultures were shaken continuously at 120 rpm on a shaker (B. Braun Biotech International, Melsungen, Germany) in a climate chamber. Changes in pH were monitored during cultivation with a pH electrode starting from ten small gametophores (<0.5 cm) in 50 mL growth medium in flasks.

For cultivation of *S. palustre* in the bioreactor, photobioreactors with 5 and 12 L working volume were used (Applikon, Schiedam, The Netherlands). For the 5 L bioreactors, the light intensity was set to 120 μmol m^−2^ s^−1^ using light tubes (Philips TLD 18 W/25) according to Hohe and Reski (2005b) at a photoperiod of 16 h light to 8 h dark. The 12 L bioreactors were illuminated with continuous light at 210 μmol m^−2^ s^−1^ with LED tubes. An adjustment of the pH was achieved by automatic titration with 0.5 M KOH and 0.5 M HCl. If the pH was not adjusted continuously, it was tracked during cultivation with an internal pH electrode. The bioreactors were aerated with 0.3 vvm air according to Hohe and Reski (2005b). The medium was used as described above, however for large scale production 2 % sucrose instead of 0.3 % sucrose were added. Before inoculating the moss in the bioreactors fresh weight was determined in laminar flow benches (AV-100, Telstar, Spain or Holten, Laminair, Thermo Scientific, Dreieich, Germany) with a scale (B502-S, Mettler Toledo, Spain or L 610 D, Sartorius, Göttingen, Germany) using a glass beaker (Simax, Sázava, Czech Republic) with a plastic filter or a Steritop^®^ filter (Millipore Corporation, Billerica, MA, USA) with a vacuum pump (Vacuubrand MZ 2C, Vacuubrand GmbH and Co, Wertheim, Germany).

### Disruption, sub-cultivation and fresh weight measurements

In order to test whether *S. palustre* growth can be enhanced by regular disruption with an Ultraturrax (Ika, Staufen, Germany), gametophores were disrupted at 4,000–18,000 rpm for 10 s up to 1 min. As this sub-cultivation technique used for example for vegetative propagation of the moss *Physcomitrella patens* (Grimsley et al. 1977) was not applicable for *S. palustre* gametophores, the peat moss cultures were disrupted manually using forceps or an autoclavable bottle (17 cm × 7 cm, Nalgene™, Thermo Scientific, Dreieich, Germany) with inert metal chicanes, i.e. screws STS-plus KN6041 5 × 30-T25 (Schriever, Lüdenscheid, Germany) by shaking the culture for 1 min within the device (Figure S1a).

For analyzing the effects of previous disruption and inoculum density on the biomass yield, comparative cultures of each two times 1, 5 and 8 g fresh weight (FW) were started in flasks containing 200 mL liquid Knop medium supplemented with ME, 0.3 % sucrose and 1.25 mM NH_4_NO_3_ (pH 4.8). One of both cultures was disrupted (Figure S1c) before cultivation by shaking the gametophores for 1 min within the device, while the other one was not disrupted (Figure S1b). After 2 and 4 weeks of cultivation the FW was determined using a scale (L 610 D, Sartorius, Göttingen, Germany). Before weighing, the gametophores were filtered for 1 min using a Steritop^®^ filter (Millipore Corporation, Billerica, MA, USA) and a vacuum pump (Vacuubrand MZ 2C, Vacuubrand GmbH and Co, Wertheim, Germany).

### Medium optimization and dry weight measurements

Growth curves were established using each ten *S. palustre* gametophores as a reference and as starting material for comparative cultivation in different growth media in Erlenmeyer flasks. Gametophores were taken from one freshly disrupted *S. palustre* culture using a specific device (Figure S1). The flasks were filled with 50 mL of the respective medium of interest. Each ten small gametophore (< 0.5 cm) were grown submerse in flasks on a shaker for 2, 4, and 6 weeks. Three replicates were done for each time point, while up to ten replicates were made to determine the initial weight of ten gametophores. For dry weight measurement after the respective cultivation time, moss material was filtered from liquid medium using sieves (Wilson Sieves, Nottingham, England). The gametophores were transferred to fresh Petri dishes under a laminar air flow clean bench (Holten, Laminair, Thermo Scientific, Dreieich, Germany) and kept there for at least 24 h under constant air flow to dry the material. Dry weight was measured with an accuracy scale (CPA 3245, Sartorius, Göttingen, Germany). For high amounts of biomass from the 12 L bioreactors, the material was dried following three consecutive drying cycles of 8 h each at 50, 80 and 100 °C in a forced air oven (Digitronic oven, Selecta, Barcelona, Spain) and weighed on a balance (B502-S, Mettler Toledo, Greifensee, Switzerland) using a plastic tray covered with filter paper (Filtros Anoia, Barcelona, Spain). The ratio of fresh to dry weight is approximately 14.0 (±2.7, n = 12).

### Light microscopy and scanning electron microscopy

For phenotypic analysis a stereo microscope (Stemi 2000-C, Zeiss, Jena, Germany) and an Axioplan microscope (Zeiss) were used. Photographs were scaled with the AxioVision software 4.8 (Zeiss). To describe the morphology of *S. palustre* grown in flasks in liquid culture, 10 shoots were randomly selected for microscopy observations. In total, five stem leaves and five divergent branch leaves were chosen per shoot. From each leaf at the middle, five chlorocystes and five hyalocystes were measured regarding length and wideness for each cell type. For the clone, thirteen shoots were weighed to determine the dry weight of one shoot in comparison to shoots from the field. Comparison of key morphological traits refers to diagnostic description and iconography according to Smith (2004). For scanning electron microscopy (SEM), *S. palustre* shoots were fixed with 3 % glutaraldehyde for 24 h at 4 °C and post-fixed in 2 % OsO_4_ in 0.1 M phosphate buffer (pH 6.8) at 4 °C for 24 h. Afterwards, shoots were thoroughly washed in phosphate buffer, cut into small pieces (3–5 mm), mounted on stubs and observed humid under an environmental SEM FEI QUANTA 200 (Fei™, Hillsboro, USA) working in extended low-vacuum (ESEM) conditions. To compare morphological traits of in vitro-cultivated *S. palustre* to plants from the field, native *S. palustre* shoots collected at Posta Fibreno Lake, southern Italy, were gently dried at room temperature and small pieces of 2–3 mm were mounted on stubs with double-sided adhesive tape and coated with carbon. Furthermore, gametophores grown on solid medium, in flasks, in aerated flasks and in the bioreactor were analyzed comparatively. The samples were analyzed under the same environmental SEM FEI QUANTA 200 working under low vacuum condition.

### Statistical analysis

To determine significance values between the single growth curves, the disruption experiments, and the effect of 0.3 and 2 % sucrose during bioreactor-based cultivation, the data were tested for normal distribution with the Kolmogorov–Smirnov test (Lilliefors 1967) followed by an analysis of variance (Fisher 1918, 1925). Afterwards, each data set was tested using a paired Students *t* test (Student 1908). The resulting p-values were corrected with Bonferroni-Holm (Holm 1979) and *p* values below 0.05 were considered to be statistically significant.

## Results

### Establishment of axenic cultures of five *Sphagnum* species

**Fig. 1:**
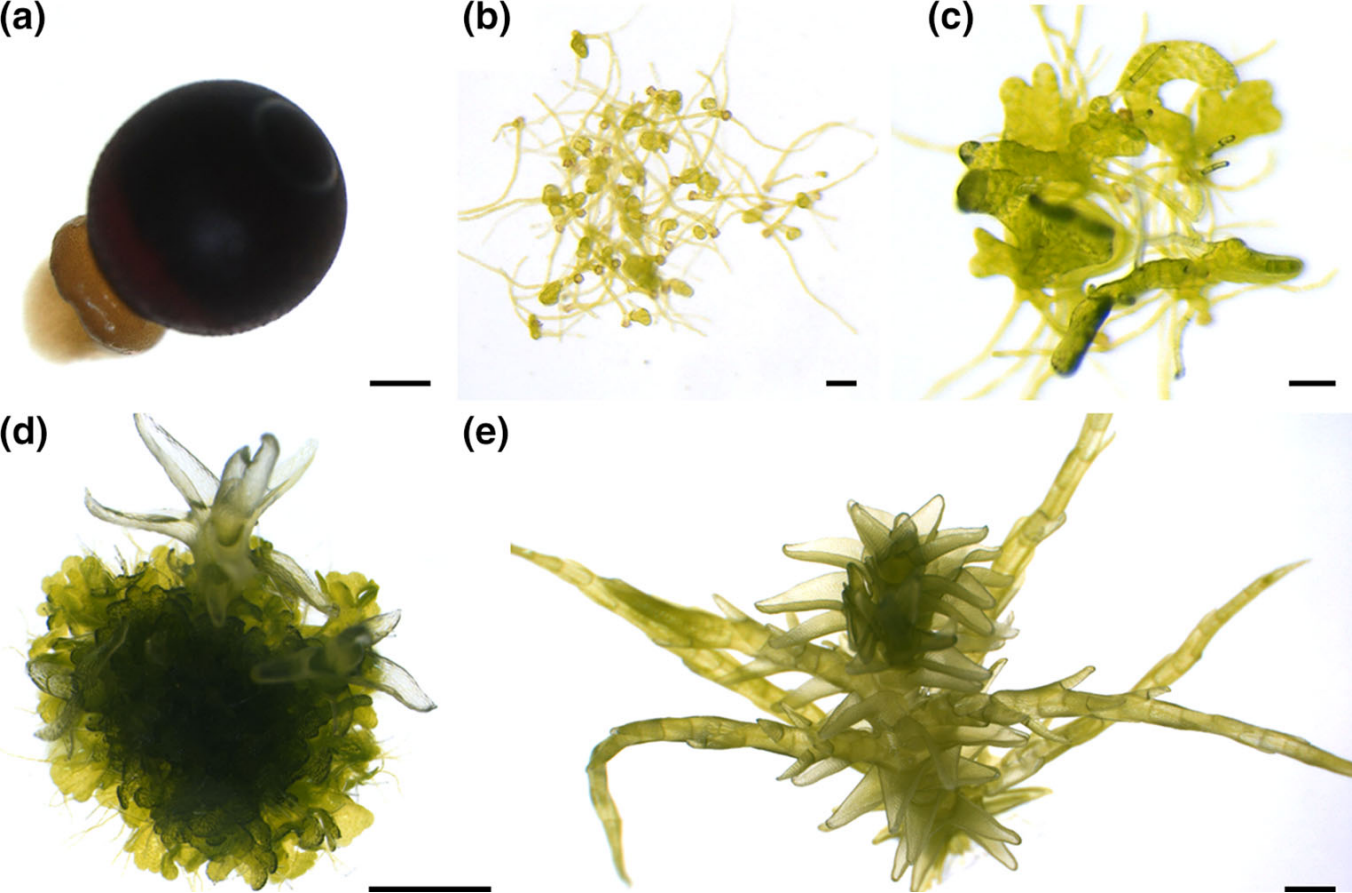
Establishment of axenic in vitro cultures of *Sphagnum palustre*. **a** *Sphagnum palustre* sporangia were collected in the field and the spores were surface sterilized, *scale bar* = 1 mm. **b** After sterilization, spores germinated within approximately 1–2 weeks and **c** filaments as well as thalloid protonema developed, *scale bars* = 0.1 mm. **d** From thalloid protonema gametophores developed, *scale bar* = 1 mm. **e** Gametophores were cultivated as independent clones and can be cultivated on solid Knop medium, *scale bar* = 1 mm

Axenic clonal cultures were established for all five *Sphagnum* species by surface sterilization of spores from mature sporangia freshly collected in the field, e.g. as shown for *S. palustre* (Fig. 1a). From surface sterilized spores, thalloid and filamentous protonema developed within 1–2 weeks (Fig. 1b, c). From thalloid protonema gametophores (Fig. 1d) developed. One gametophore (Fig. 1e) was regarded as single clone and further cultivated. In total, ten independent clones of each species were further propagated. One clone of *S. palustre* was randomly selected and chosen for further analyses and large-scale biomass production for biomonitoring. One clone of each species is stored in the International Moss Stock Center (IMSC, http://www.moss-stock-center.org). The corresponding IMSC numbers are 40068 (*S. palustre*), 40069 (*S. fimbriatum*), 40066 (*S. magellanicum*), 40067 (*S. rubellum*), and 40070 (*S. subnitens*).

**Fig. 2:**
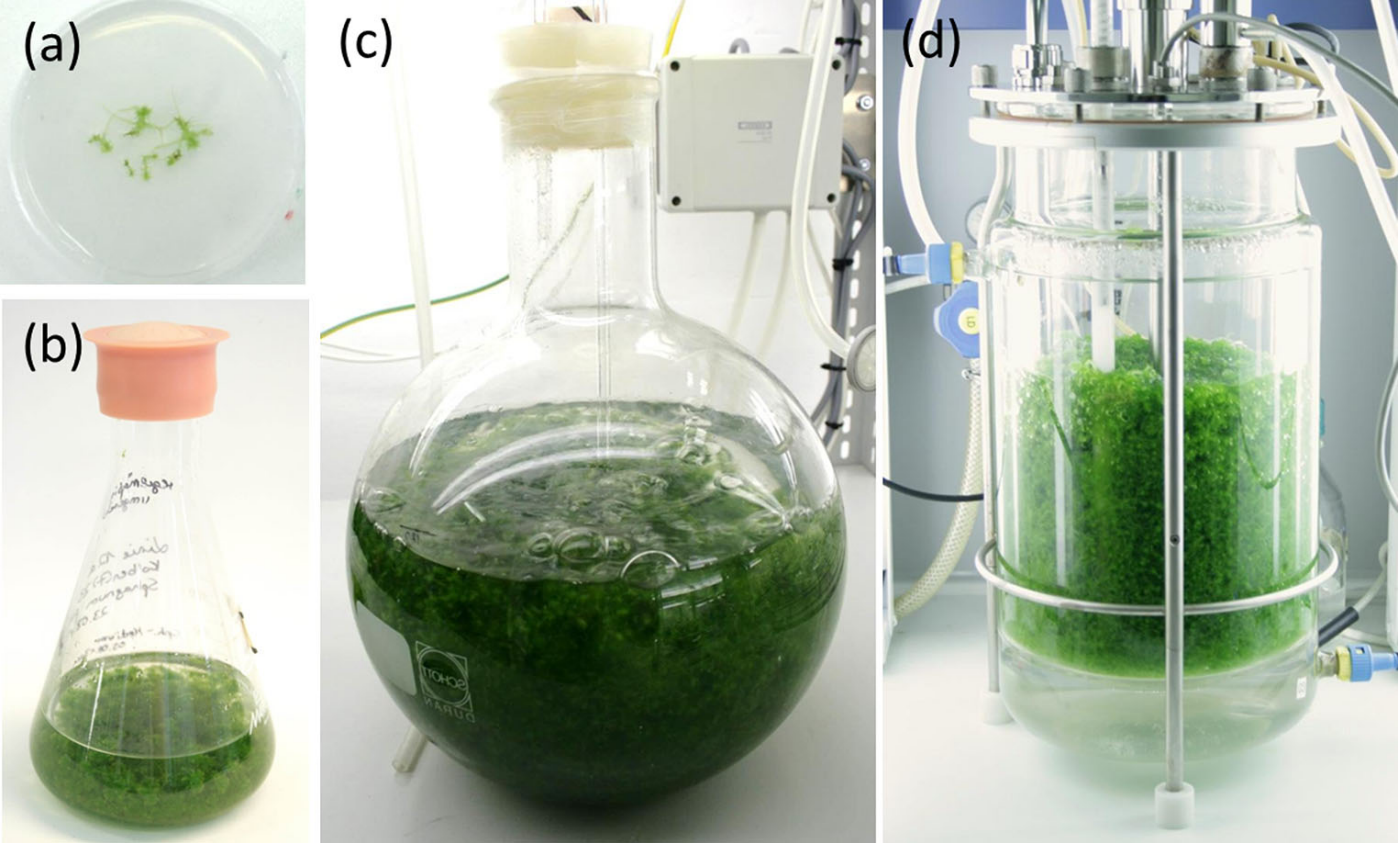
Cultivation techniques for *Sphagnum palustre*. Gametophores can be cultivated on **a** solid Knop medium on Petri dishes, **b** in Erlenmeyer flasks, **c** in 5 L aerated flasks and **d** in a photobioreactor containing liquid Knop medium with microelements supplemented with 0.3 % sucrose and 1.25 mM ammonium nitrate

In order to establish in vitro cultivation procedures and to produce high quantities of *S. palustre*, we tested different cultivation techniques, optimized the growth medium and analyzed the effect of disruption and inoculum density on the growth of the species. *S. palustre* gametophores can be grown on solid Knop medium with and without ME in Petri dishes (Fig. 2a). Due to the comparatively slow growth, this method is suitable for long-term storage. Furthermore, *S. palustre* gametophores can be cultivated in suspension culture either in flasks (Fig. 2b), 5 L aerated flasks (Fig. 2c) or in a photobioreactor (Fig. 2d).

**Fig. 3:**
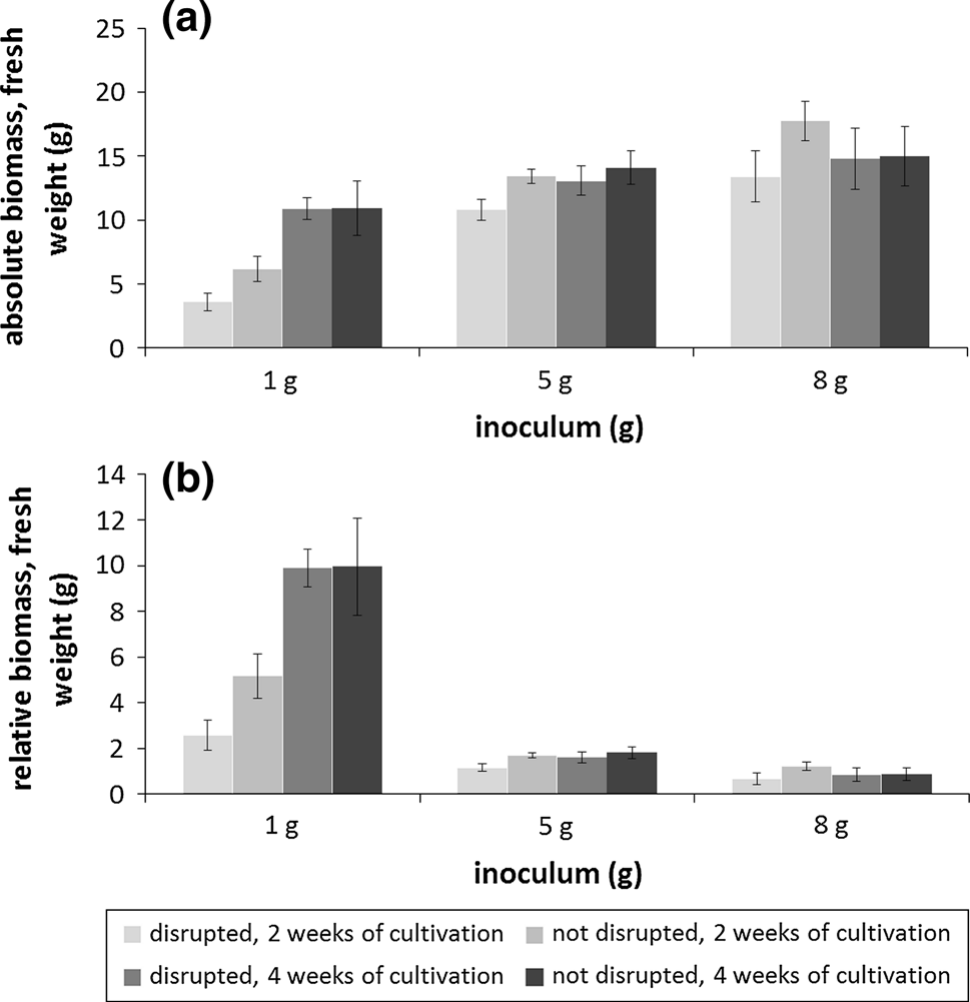
The effect of inoculum and disruption on biomass yield. *Sphagnum palustre* gametophores were cultivated in flasks filled with 200 mL liquid medium (Knop, microelements, 1.25 mM ammonium nitrate, 0.3 % sucrose). As inoculum either 1, 5 or 8 g fresh weight (FW) were used. Undisrupted gametophore material was cultivated in parallel and in comparison to gametophore material that has been disrupted with a specific device, an autoclavable screw-cap plastic can with 20 inert metal chicanes (for details see Figure S1), before cultivation. After 2 and 4 weeks the FW was measured. **a** Absolute biomass yield of disrupted and undisrupted material after 2 weeks (*light grey bars*) and 4 weeks (*dark grey bars*) of cultivation. The amount of FW used as inoculum is shown on the x-axis, the absolute biomass is shown on the y-axis. **b** Relative biomass yield of disrupted and undisrupted material after 2 weeks (*light grey bars*) and 4 weeks (*dark grey bars*) of cultivation. The amount of FW used as inoculum is shown on the x-axis, the relative biomass is shown on the y-axis

Cultivation of pure protonema suspension culture was not achieved yet, as most material of *S. palustre* gametophores died after disruption with an Ultraturrax as indicated by brownish moss material after 2 weeks (Figure S2). Nevertheless, disruption with a specific device was possible but yielded no higher amounts of biomass within 2 and 4 weeks of cultivation (Fig. 3). In contrary, gametophores that were disrupted before cultivation produced significantly less biomass after 2 weeks for all three analyzed inocula of 1, 5 and 8 g fresh weight (FW), while this effect was compensated after 4 weeks (Fig. 3a). The highest relative biomass was obtained with an inoculum of 1 g (Fig. 3b).

### Sucrose supplementation enhances biomass yields

**Fig. 4:**
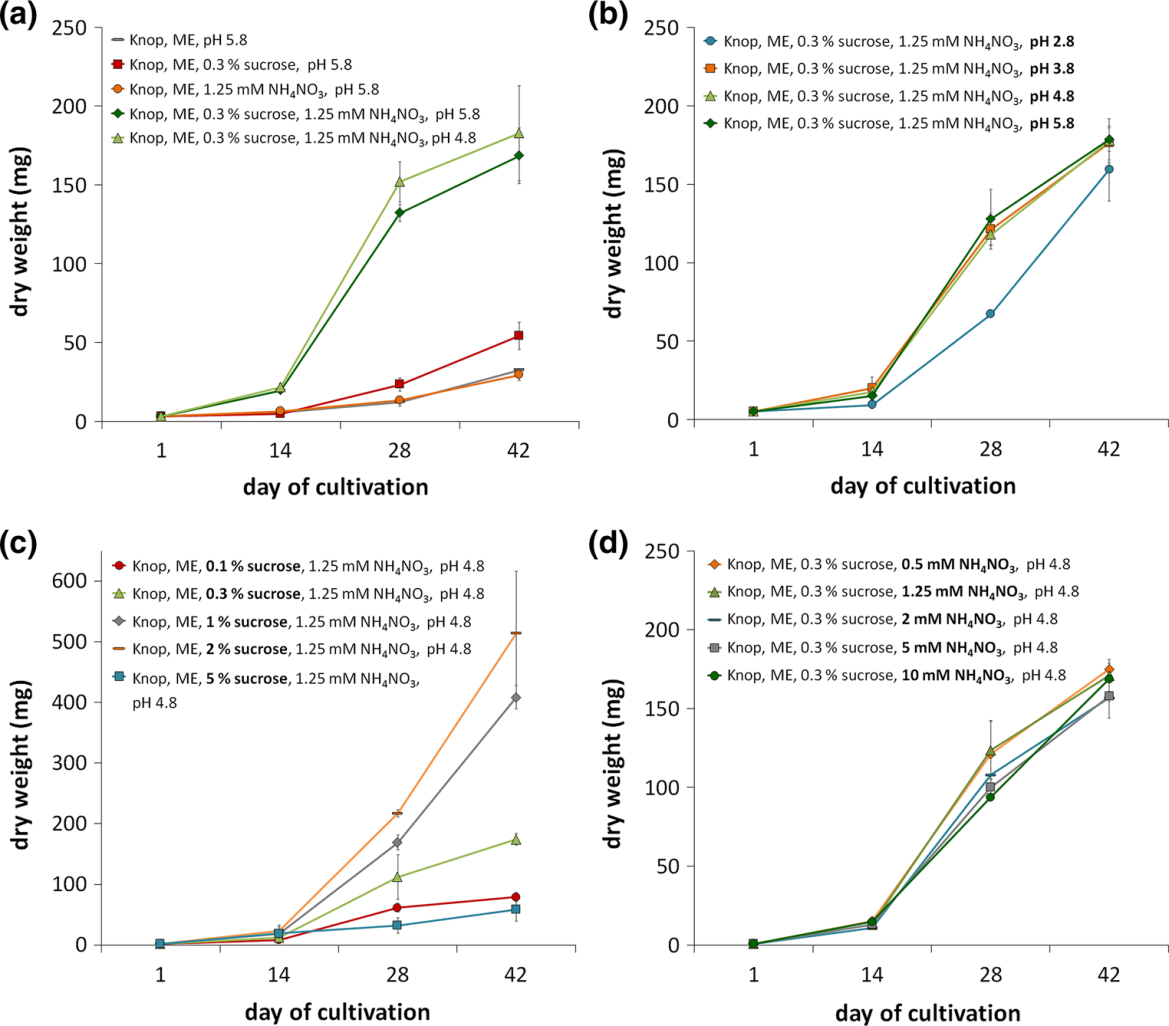
Biomass increase of *Sphagnum palustre* grown in different cultivation media. *Sphagnum palustre* gametophores were cultivated in flasks each containing 50 mL of different media to be tested. **a** A comparative study was done to analyze the biomass increase in Knop with ME, Knop with ME and 0.3 % sucrose, Knop with ME and 1.25 mM NH_4_NO_3_, and Knop with ME, 0.3 % sucrose and 1.25 mM NH_4_NO_3_; the latter at two different starting pH of 4.8 and 5.8. **b** Knop with ME, 0.3 % sucrose and 1.25 mM NH_4_NO_3_ with different starting pH of 2.8, 3.8, 4.8, and 5.8 were tested. **c** Knop with ME, 1.25 mM NH_4_NO_3_ with different concentrations of 0.1, 0.3, 1, 2 and 5 % sucrose were compared. **d** Knop with ME, 0.3 % sucrose and different NH_4_NO_3_ contents were analyzed. In case only one parameter was exchanged this is highlighted in *bold*. The y-axis shows the dry weight, the x-axis shows the day of cultivation

For optimization of biomass yield, the cultivation medium was optimized regarding its composition, the pH, the sucrose content and the ammonium nitrate (NH_4_NO_3_) content (Fig. 4). Starting from Knop medium with ME we added either 0.3 % sucrose or 1.25 mM NH_4_NO_3_, as well as 0.3 % sucrose and 1.25 mM NH_4_NO_3_ at the standard pH of 5.8 and a lower pH of 4.8 (Fig. 4a). While addition of 0.3 % sucrose alone as well as 1.25 mM NH_4_NO_3_ alone yielded no or not highly increased biomass, supplementation with both, 0.3 % sucrose and 1.25 mM NH_4_NO_3_, yielded significantly increased amounts of biomass after 2, 4, and 6 weeks of cultivation (Fig. 4a). The starting pH of 4.8 and 5.8 yielded comparable amounts of biomass within 6 weeks, however with slightly, but not significantly more biomass using a pH of 4.8. To analyze whether a lower pH was even better, different starting pH were tested, showing that there are no significant differences in biomass increase between pH 3.8 and pH 5.8, while a pH of 2.8 is unsuitable for fast growth (Fig. 4b). After 6 weeks of cultivation, the medium with a starting pH of 2.8 also showed comparable results as the other media (Fig. 4b).

In contrast to the concentration of NH_4_NO_3_ (Fig. 4d), the concentration of sucrose strongly affects the biomass increase of *S. palustre* (Fig. 4c). Supplementation with 2 % sucrose resulted in the highest biomass increase after 4 and 6 weeks (Fig. 4c). In all analyzed media, a lag phase of approximately 2 weeks was observed. Summing up, we propose using Knop medium with ME, 1.25 mM NH_4_NO_3_ and, depending on the required biomass increase, 0.3–2 % sucrose for cultivation of *S. palustre* gametophores in liquid medium.

### Optimized in vitro cultivation of *Sphagnum palustre* in bioreactors

When cultivating *S. palustre* in the photobioreactor (Fig. 2d), we observed that neither disruption with a stirrer at 500 rpm nor maintenance of a fixed pH was suitable for the cultivation of this species. An adjustment of the pH (pH 5.0, n = 3) yielded cloudy medium and material with increased amounts of white tissue, while disruption caused higher amounts of brownish material.

In consequence, we cultivated *S. palustre* in the bioreactor under aerated conditions starting with pH 4.0, according to the pH of the commonly used growth medium after autoclaving, without regulating it during the following cultivation process. Using this technique, we gained between 320 and 470 g FW within 4–5 weeks (n = 4) when starting with around 15 g FW in 5 L bioreactors using 0.3 % sucrose in the growth medium. By addition of 2 % sucrose to the medium up to 500 g FW were obtained within 3–4 weeks in 5 L bioreactors when starting with the same amount of moss. Depending on the sucrose concentration, comparable biomass increases were achieved in 12 L bioreactors. When comparable starting dry weights of 3.0 g ± 0.5 were used in bioreactors with each 0.3 % (3.6 g ± 0.3, n = 7) and 2 % (3.8 g ± 0.2, n = 5) sucrose-containing medium, the final dry weight was significantly higher when 2 % sucrose was used (117 g ± 16, n = 5) instead of 0.3 % sucrose (41 g ± 7, n = 7), while the time of cultivation was decreased slightly from 31 ± 1.8 days using 0.3 % sucrose to 29 ± 3.6 days using 2 % sucrose (Table S1).

### *Sphagnum palustre* changes the pH of the medium during cultivation

**Fig. 5:**
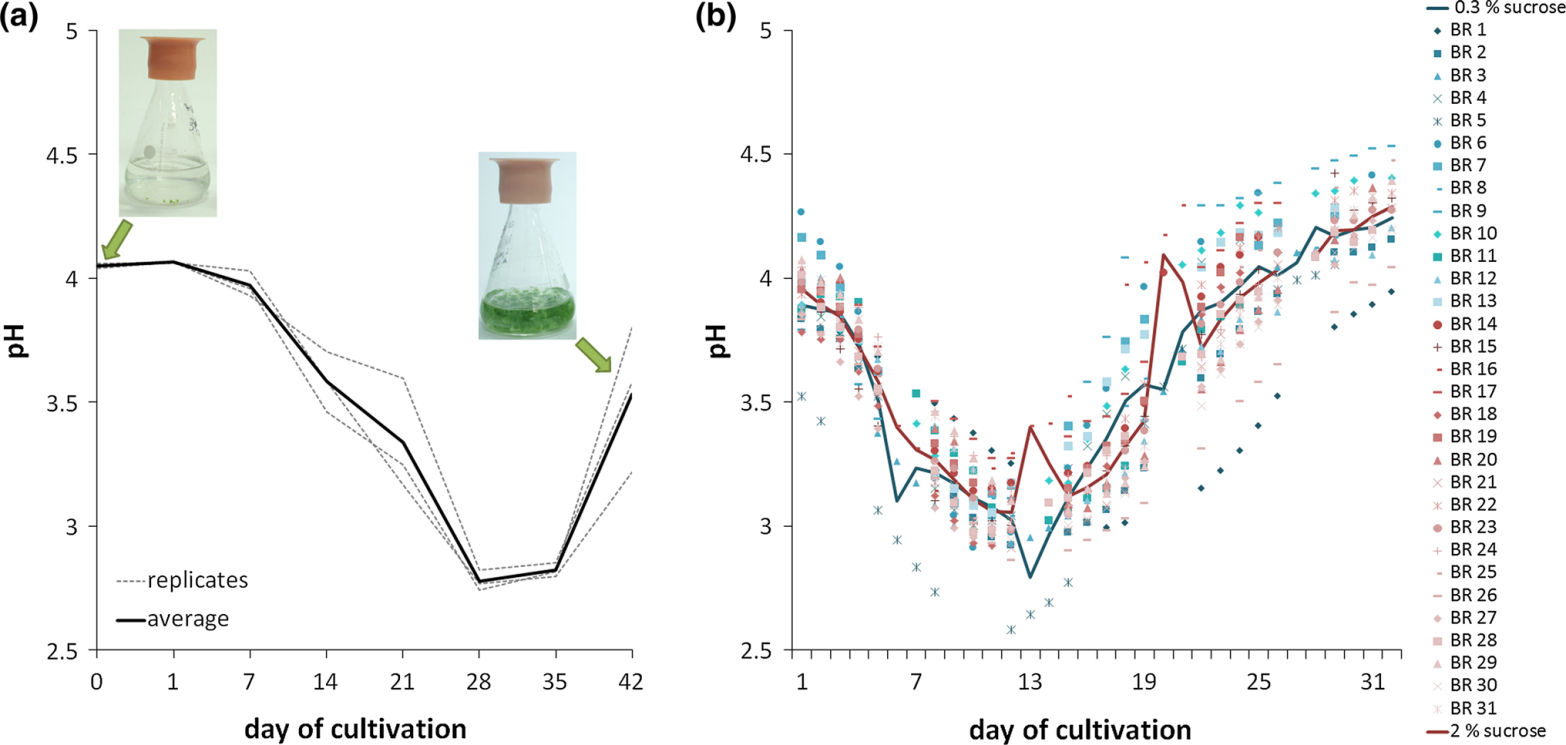
Changes in pH during in vitro cultivation of *Sphagnum palustre*. *S. palustre* acidifies the medium during cultivation. The y-axis shows the pH, while the x-axis shows the day of cultivation. **a** Ten *S. palustre* gametophores cultivated in flasks with 50 mL growth medium (Knop, microelements, 0.3 % sucrose, 1.25 mM ammonium nitrate) acidify the medium from pH 4 to pH 2.8 (±0.04) within 28 days. Afterwards, the pH increases again. *Black line* shows the average of the pH, *grey dotted lines* show the three replicates (independent flasks and measurements). **b** Also during cultivation in 12 L bioreactors the pH first decreases and increases again. In diverse symbols the pH from cultures grown in independent bioreactors is shown, while the *lines* show the average of the pH from independent cultivation processes; *blue* = 0.3 % sucrose, *red* = 2 % sucrose

It is well characterized that peat mosses acidify their natural environment. To analyze the effect of ion exchange during *S. palustre* cultivation in vitro, we monitored the pH of the cultivation medium from flasks and from the 12 L bioreactor. In fact, *S. palustre* acidifies its growth medium during the first weeks of cultivation (Fig. 5). Ten *S. palustre* gametophores cultivated in 50 mL growth medium-containing flasks acidify the medium from pH 4 to pH 2.8 (±0.04, n = 3) within 28 days (Fig. 5a). During the cultivation process the pH increases again. The same effect was observed in the bioreactor-based cultivation. As initial material tissue equivalent to 1–6 mg dry weight was transferred to 12 L bioreactors and the pH was measured regularly (Table S1). Using 0.3 % sucrose, the pH decreases from initial 3.89 (±0.18) to 2.80 (±0.22) and 2.97 (±0.2) within 2 weeks (Fig. 5b). Using 2 % sucrose, the pH changes are very comparable to the cultivation with 0.3 % sucrose, showing no significantly different trend, while biomass increased significantly faster (Table S1).

### Morphological characterization of in vitro-cultivated *Sphagnum palustre*

**Fig. 6:**
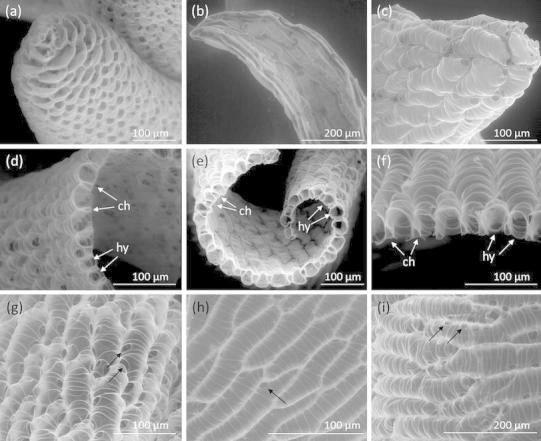
Morphological characterization of *Sphagnum palustre* with scanning electron microscopy under low vacuum mode. **a** Cucullate leaf apex of the field sample in comparison to **b** lanceolate leaf apex of the clone grown in flasks and **c** cucullate leaf apex of clone grown on solid medium. **d** Leaf section of the field sample and of clone **e** grown in flasks and **f** in the bioreactor. Chlorocystes (ch) and hyalocystes (hy) are indicated with *white arrows*. Abaxial surface of a leaf from (**g**) the field sample and from (**h**, **i**) the clone grown in flask, (**h**) once with small and very few pores and (**i**) once with many pores comparable to the field sample. Pores are indicated with *black arrows*

The main morphological characteristics distinguishing in vitro-cultivated *S. palustre* from native *S. palustre* shoots (Posta Fibreno, Latium, Italy) are summarized in Table 1. In general, the field shoots were thicker and heavier than those from in vitro-grown material. The average dry weight of field samples was 14.76 mg (±7.2, n = 10), while the in vitro-cultivated material had a weight of 2.72 mg (±1.13, n = 13). The length of leaves was comparable in both samples, however, as analyzed by SEM (Fig. 6), field shoots have cucullate, ovate leaves (Fig. 6a), while lanceolate, not cucullate leaves were observed in the clone grown in flasks (Fig. 6b). Lanceolate leaves were also observed from material cultivated in aerated flasks and the bioreactor, while both, cucullate and lanceolate leaves were observed from material grown on solid medium (Fig. 6c). Field leaf section highlights a mid-lamina thickness of 30–50 µm across the hyalocystes, and 19–30 µm across the chlorocystes, with a hyalocystes to chlorocystes wideness ratio of approximately 5 or higher in the field sample (Fig. 6d). Clone leaf sections showed a quite variable thickness of approximately 25–40 µm across the hyalocystes, and 7–18 µm across the chlorocystes, with a hyalocystes to chlorocystes wideness ratio of about 2–5.5 (Figs. 6e, f). In general, this seemed to be a highly variable morphological trait, as larger but also smaller chlorocystes in relation to the hyalocystes were observed. Another quite variable morphological characteristic was the number of pores per hyalocyste. While 3–16 pores were observed in material from the field (Fig. 6g), the number of pores was often found to be 2–3 in the clone (Fig. 6h). However, also comparable numbers of pores per hyalocyste were detected in the clone (Fig. 6i). The morphology of *S. palustre* from in vitro cultivation was in general quite variable regarding the size of the cells or the number of pores per hyalocyste. Clear differences in morphology related to the different cultivation techniques (Fig. 2) were not detected.

**Table 1:** Morphological characterization of *Sphagnum palustre* from in vitro cultures and material from the field

Tissue or cell types	Clone		Field shoot (FS)***	
	Length (µm)	Wideness (µm)	Length (µm)	Wideness (µm)
Stem leaves*	1,756 ± 532	731 ± 383	1,600	1,150
Branch leaves*	1,969 ± 497	715 ± 346	2,250	1,450
Hyalocystes**	146 ± 47	27 ± 9	338	50
Chlorocystes**	115 ± 43	11 ± 4	62	9
Dry weight (mg)	2.72 ± 1.13^#^		14.76 ± 7.20^##^	
Pore diameter (µm)	5–8		10–25	

* Mean value of 50 measures ± standard deviation (SD), ** mean value of 250 measures ± SD, *** Field shoots (average values from Smith 2004), ^#^ mean value of 13 shoots ± SD, ^##^ mean value of 10 shoots ± SD

## Discussion

Moss clones from in vitro culture share the same genetic, physiological and environmental background as they were established from one single spore and cultivated under the same growth conditions. They provide the potential to serve as model organisms for a broad spectrum of molecular, but also evolutionary-developmental questions. As proved for the model organism *P. patens*, strains available in axenic in vitro culture facilitate basic as well as applied research. Like no other moss, *P. patens* is nowadays studied on metabolomic, proteomic and transcriptomic level, and provides novel insights into genome as well as land plant evolution (Erxleben et al. 2012; Mueller et al. 2014, Beike et al. 2014a). Considering their morphological, developmental and biochemical characteristics on the one hand, and their ecological and economic relevance on the other hand, *Sphagnopsida* are very interesting organisms for basic as well as applied research. As scalable in vitro cultures improve the availability of species of interest for research and application, the establishment of five different *Sphagnum* species in axenic culture represents an important resource for future studies.

The biomass increase obtained for *S. palustre* under optimized in vitro conditions is much higher than reported from the field. Within 4 weeks, we were able to increase the biomass of *S. palustre* by around 30-fold in the bioreactor and, depending on the inoculum, up to tenfold in flasks. Growth experiments for *Sphagnum* farming with *S. palustre* in the field in Northern Germany showed a biomass increase by 2.5-fold within 1 year (cf. Gaudig et al. 2014). These results substantiate that in vitro cultivation with this high multiplication rate could supply sufficient peat mosses as ‘seeding’ material for installation of 40,000 ha *Sphagnum* fields on degraded peatlands, which would be necessary to substitute slightly humified peat for horticultural substrates in Germany (Krebs et al. 2014). Furthermore, the use of in vitro-cultivated material represents a worthwhile alternative to the collection of material from the field for basic research, especially for peat mosses that are protected by laws.

In previous in vitro cultivation studies on *Sphagnum*, growth-promoting effects of sucrose or glucose-addition have been shown, while growth without addition of an organic carbon source was rather slow (Simola 1969; Kajita et al. 1987; Rudolph et al. 1988; Graham et al. 2010). Our study showed that, also for *S. palustre*, cultivation with an additional carbon source is favorable, revealing an optimal sucrose concentration of 2 %. Without addition of an external carbon source the growth was very slow, which is a clear difference to *P. patens* that does not require an external carbon source for fast growth in vitro. However, also for this species, the growth can be increased by CO_2_ addition (Decker and Reski 2008). Our results, together with results obtained from other peat moss in vitro studies (Simola 1969; Kajita et al. 1987; Rudolph et al. 1988; Graham et al. 2010) provide further evidence for the widespread ability of peat mosses to take up and use exogenous carbon in form of sugars (mixotrophy).

Considering the growth experiments of *S. palustre* in flasks, we infer that at a certain density of gametophores per volume and duration of cultivation, nutrient or light availability of this photomixotrophic culture might have been a limiting factor. This could be an explanation for the high relative biomass increase using a small inoculum and the low relative biomass increase when more material was used as inoculum. Correspondingly, the absolute biomass after 4 weeks was for all cultures in a comparable range disregarding the amount of starting material.

Once the cultivation of *S. palustre* in flasks and bioreactors was started, the pH starts to decrease. Acidification by *Sphagnopsida* has been reported from the natural environment (Clymo 1963, 1964), but also for in vitro cultures in a continuous feed fermenter (Rudolph et al. 1988). As shown by pH measurements and comparative growth experiments, *S. palustre* also acidifies the growth medium, a fact that is related to ion exchange, i.e. hydrogen ion release, at the surface of the plants in order to take up cations (Clymo 1963, 1964). The carboxylic and phenolic groups of the cell walls have been suggested to be the main proton-binding sites on the surface of *Sphagnum* species (0.65 mmol/g) responsible for efficient adsorption of metals on moss (González and Pokrovsky 2014). In consequence, a starting pH between 3.8 and 5.8 showed no severe impact on the growth as it was changed by the plants during cultivation. Considering the growth curves, the lag phase during the first 2 weeks might be connected with ion exchange and the development of apices.

Another feature of *S. palustre* cultivation that is quite different from cell culture techniques used for the model organism *P. patens*, is the high sensitivity of its gametophores towards disruption with an Ultraturrax. This procedure is performed for *P. patens* to grow standardized, homogenized protonema suspension cultures. Obviously, differentiated *S. palustre* gametophores do not have such a high regeneration capacity like *P. patens* cells where eight different types of stem cells have been identified (Kofuji and Hasebe 2014). Gametophores of *Sphagnum* are described to have a strictly determined organography with an apex of almost unlimited growth that produces stem and branch primordia, the latter organized in fascicles (Clymo and Duckett 1986). Regeneration experiments of *S. magellanicum*, *Sphagnum papillosum* Lindb. and *Sphagnum recurvum* Palisot de Beauvois revealed that over 90 % of new shoots emerge from the immediate vicinity of branches and fascicles (Clymo and Duckett 1986). For *S. palustre*, it was shown that green parts of the stems and apical branches have highest regeneration ability, while leaves did not regenerate to develop new gametophores (Sobotka 1976). A strict localization of stem cell activity is a possible explanation of why manual disruption with forceps or chicanes was possible, while complete disruption with an Ultraturrax was lethal for the majority of the gametophores. Although an advantage of previous disruption on biomass increase for longer cultivation times cannot be excluded at this point, we do not see clear advantages of disruption within the cultivation time of 4 weeks that was applied here.

Phenotypic analyses of the in vitro-cultivated *S. palustre* clone showed considerable variations in morphology, e.g. with regard to the number of pores per hyalocyste, leave shape or cell sizes. As summarized by Christy (1987), morphological characters of bryophytes, including their general size, show a high variability during cultivation. The in vitro-grown *S. palustre* gametophores were in general smaller in size, while showing comparable morphological characteristics as the material collected from the field. This would indicate a higher surface-to-mass ratio of the clonal material as a possible advantage for biomonitoring purposes. In consequence, the analysis of pollutant accumulation capacity will be the next step towards standardized air quality measurement using peat moss bags.

## Electronic supplementary material

Below is the link to the electronic supplementary material.

Supplementary material Tissue disruption with a specific device. (a) An autoclavable screw-cap plastic can (17 cm x 7 cm, Nalgene™, Thermo Scientific, Dreieich, Germany) with 20 inert metal chicanes, i.e. screws STS-plus KN6041 5x30-T25 (Schriever, Lüdenscheid, Germany) was used for manual disruption of *Sphagnum palustre* gametophores. The moss was shaken within the device for 1 min. (b) Overview on *S. palustre* gametophore material before disruption and (c) after disruption using this device, scale bar = 1 cm (TIFF 18357 kb)

Supplementary material Disruption of *Sphagnum palustre* gametophore cultures with an Ultraturrax*. Sphagnum palustre* gametophore cultures were disrupted with an Ultraturrax. (a) The tissue was disrupted for 1 min at 18,000 rpm. After 2 weeks the material was browned. (b) Gametophores were disrupted for 10 s at 18,000 rpm. After 2 weeks the disrupted material was browned. (c) One culture was disrupted for 20 s at 4,000 rpm. Again, the material was brown after 2 weeks (TIFF 24929 kb)

Supplementary material 3 (XLS 69 kb)
