# Supplementary figures and images for: Clonal in vitro propagation of peat mosses (Sphagnum L.) as novel green resources for basic and applied research
Source: Plant Cell Tissue Organ Cult. 2014 Nov 14;120(3):1037–49. doi: 10.1007/s11240-014-0658-2 (PMC4551280; doi:10.1007/s11240-014-0658-2)

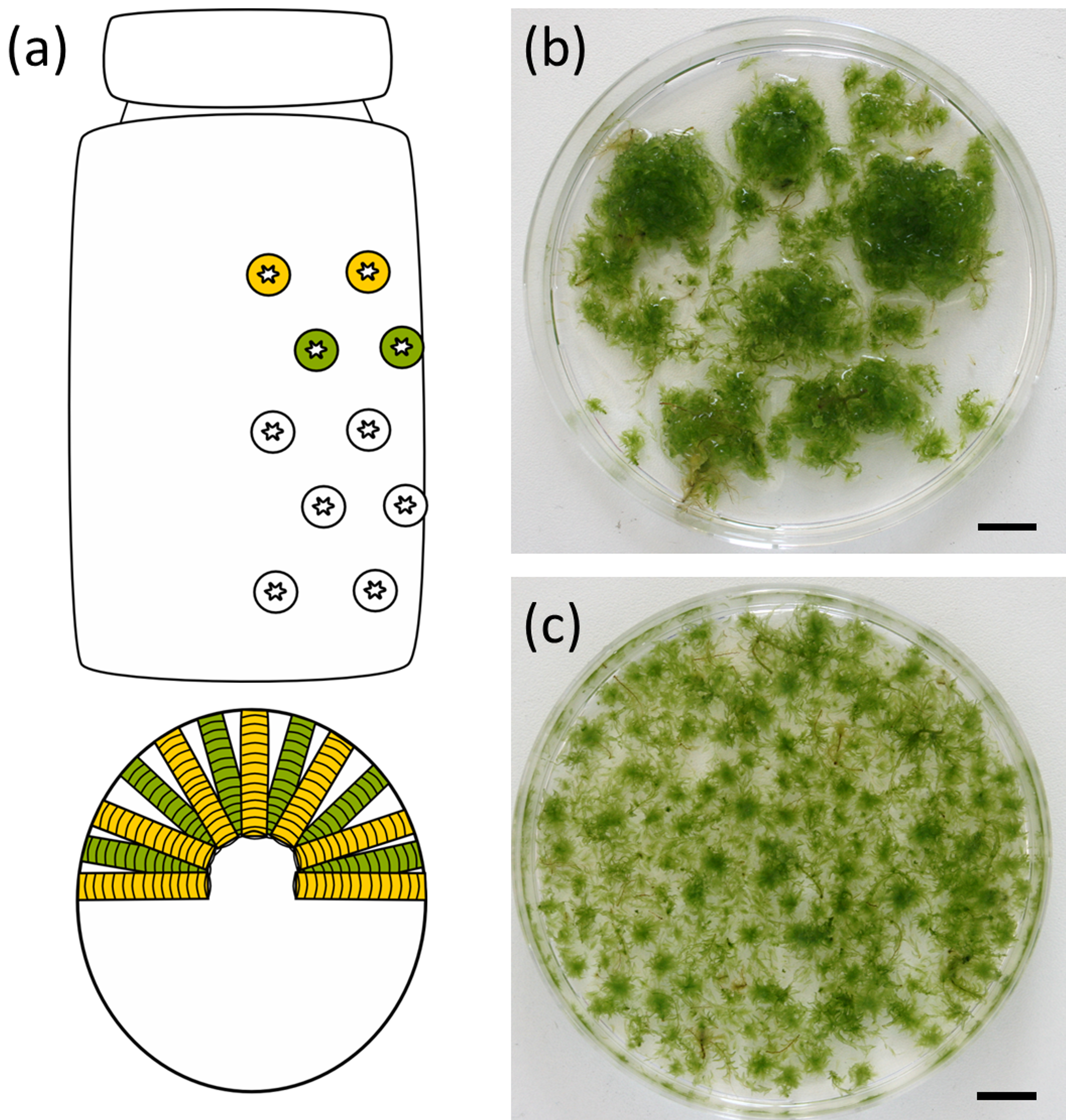

Supplement: Supplementary file 1 — Supplementary material Tissue disruption with a specific device. (a) An autoclavable screw-cap plastic can (17 cm x 7 cm, Nalgene™, Thermo Scientific, Dreieich, Germany) with 20 inert metal chicanes, i.e. screws STS-plus KN6041 5x30-T25 (Schriever, Lüdenscheid, Germany) was used for manual disruption of Sphagnum palustre gametophores. The moss was shaken within the device for 1 min. (b) Overview on S. palustre gametophore material before disruption and (c) after disruption using this device, scale bar = 1 cm (TIFF 18357 kb) [file 11240_2014_658_MOESM1_ESM.tif]

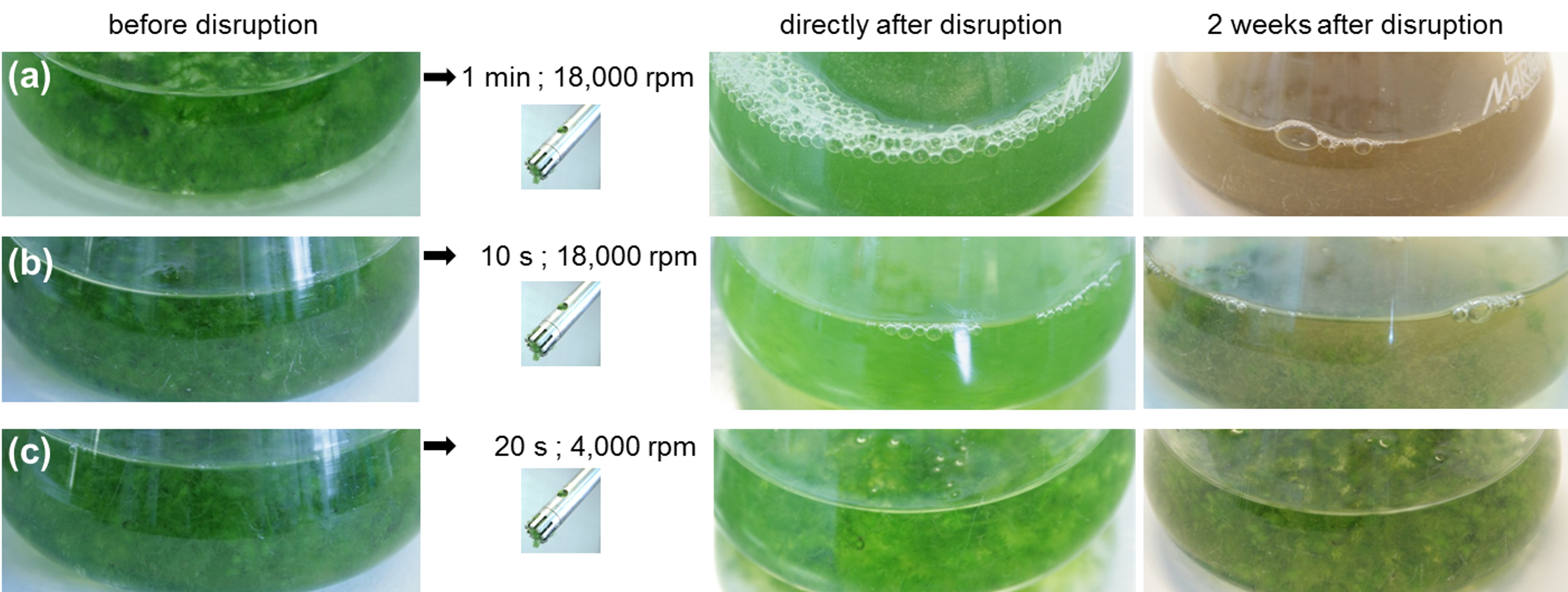

Supplement: Supplementary file 2 — Supplementary material Disruption of Sphagnum palustre gametophore cultures with an Ultraturrax. Sphagnum palustre gametophore cultures were disrupted with an Ultraturrax. (a) The tissue was disrupted for 1 min at 18,000 rpm. After 2 weeks the material was browned. (b) Gametophores were disrupted for 10 s at 18,000 rpm. After 2 weeks the disrupted material was browned. (c) One culture was disrupted for 20 s at 4,000 rpm. Again, the material was brown after 2 weeks (TIFF 24929 kb) [file 11240_2014_658_MOESM2_ESM.tif]
